# Identification and Comprehensive Analysis of FREM2 Mutation as a Potential Prognostic Biomarker in Colorectal Cancer

**DOI:** 10.3389/fmolb.2022.839617

**Published:** 2022-02-18

**Authors:** Hanpeng Du, Haiyue Wang, Fandong Kong, Mingjian Wu, Wei Chen, Jin Lyu, Sitong Zhou, Ronghua Yang

**Affiliations:** ^1^ Department of Gastrointestinal Surgery, Panyu Maternal and Child Care Service Centre of Guangzhou (He Xian Memorial Affiliated Hospital of Southern Medical University), Guangzhou, China; ^2^ Department of Nutrition, The First Hospital of Hebei Medical University, Shijiazhuang, China; ^3^ Department of Pancreaticobiliary Surgery, The First Affiliated Hospital of Sun Yat-sen University, Guangzhou, China; ^4^ Department of Pathology, The First People’s Hospital of Foshan, Foshan, China; ^5^ Department of Dermatology, The First People’s Hospital of Foshan, Foshan, China; ^6^ Department of Burn and Plastic Surgery, Guangzhou First People’s Hospital, School of Medicine, South China University of Technology, Guangzhou, China

**Keywords:** Frem2, gene mutation, colorectal cancer, prognosis, biomarker

## Abstract

Gene mutations play an important role in tumor progression. This study aimed to identify genes that were mutated in colorectal cancer (CRC) and to explore their biological effects and prognostic value in CRC patients. We performed somatic mutation analysis using data sets from The Cancer Genome Atlas and International Cancer Genome Consortium, and identified that *FREM2* had the highest mutation frequency in patients with colon adenocarcinoma (COAD). COAD patients were divided into *FREM2*-mutated type (*n* = 36) and *FREM2*-wild type (*n* = 278), and a Kaplan-Meier survival curve was generated to perform prognostic analysis. A *FREM2*-mutation prognosis model was constructed using random forest method, and the performance of the model was evaluated using receiver operating characteristic curve. Next, the random forest method and Cox regression analysis were used to construct a prognostic model based on the gene expression data of 36 *FREM2*-mutant COAD patients. The model showed a high prediction accuracy (83.9%), and 13 prognostic model characteristic genes related to overall survival were identified. Then, the results of tumor mutation burden (TMB) and microsatellite instability (MSI) analyses revealed significant differences in TMB and MSI among the risk scores of different prognostic models. Differentially expressed genes were identified and analyzed for functional enrichment and immune infiltration. Finally, 30 samples of CRC patients were collected for immunohistochemical staining to analyze the FREM2 expression levels, which showed that FREM2 was highly expressed in tumor tissues. In conclusion, CRC patients had a high level of *FREM2* mutations associated with a worse prognosis, which indicated that *FREM2* mutations may be potential prognostic markers in CRC.

## Introduction

Colorectal cancer (CRC) is one of the most common malignant tumors that seriously endanger human health nowadays (Sung et al., 2021). In recent years, changes in dietary structure and living habits have been accompanied by an increase in the incidence and mortality of CRC patients in China (Feng et al., 2019). Nonetheless, the prognosis of CRC patients remains remarkably poor, highlighting the need for further understanding the molecular mechanism of the development of CRC and the identification of new prognostic biomarkers. The pathogenesis of CRC is complex and involves genetic and environmental factors. Previous studies have found that gene mutations leading to abnormal cell signal transduction are closely related to the occurrence and development of CRC (Nakayama and Oshima, 2019).

*FRAS1 Related Extracellular Matrix 2 (FREM2)*, located on 13q13.3, encodes an integral membrane protein that contains a large amount of chondroitin sulfate proteoglycan element repeats and Calx-beta domains (Yu et al., 2018), which confer it with sodium-calcium exchanger activity, permitting this protein to export calcium from the cell. Additionally, FREM2 forms part of the FREM2-FRAS1-FREM1 protein complex, which plays an important role in epidermal-dermal interactions (Kiyozumi et al., 2006). Previous studies have found that FREM2 is related to the development of the eye (Zhang et al., 2019) and kidney epithelium (Al-Hamed et al., 2021). Recently, it has been found that FREM2 is highly expressed in gliomas and that patients with high expression levels of FREM2 show a better prognosis (Jovcevska et al., 2019). However, the role of FREM2 in CRC has not been investigated to date.

In the study, we performed somatic mutation analysis using The Cancer Genome Atlas (TCGA) and International Cancer Genome Consortium (ICGC) databases and we found that *FREM2* had the highest mutation frequency. First, prognostic analysis revealed that CRC patients with *FREM2* mutations had a worse prognosis. Subsequently, a prognostic model was constructed based on the gene expression data of 36 *FREM2*-mutant CRC patients, the efficacy of the model was evaluated, and 13 prognostic model characteristic genes related to OS were identified. Next, the tumor mutation burden (TMB) and microsatellite instability (MSI) were compared between the risk scores of different prognostic models. The genes differentially expressed between *FREM2*-mutant type and *FREM2-*wild type were identified, and functional enrichment and immune infiltration analysis were performed. Finally, the FREM2 protein expression levels were detected using immunohistochemical staining in 30 CRC patient tissues. In conclusion, *FREM2* was highly expressed in CRC and showed a higher level of mutation in CRC patients than in healthy controls. The presence of *FREM2* mutations was associated with a worse prognosis in CRC patients, indicating that *FREM2* mutation may be a potential prognostic biomarker for CRC.

## Materials and Methods

### Data Processing

Gene somatic mutation data (MAF files) were downloaded from the colon adenocarcinoma (COAD) project of TCGA (http://cancergenome.nih.gov/) (Tomczak et al., 2015) and COAD-CN cohorts of ICGC (www.icgc.org). RNAseq data in level 3 HTSeq-FPKM format was downloaded from TCGA-COAD. The RNAseq data in fragments per kilobase per million (FPKM) format was converted into transcripts per million reads (TPM) format and log2 conversion was performed for subsequent analysis. The main goal of the ICGC database is to comprehensively study the genomic changes in a variety of cancers that contribute to the global burden of human disease. It comprises data on about 50 different cancer types (or subtypes), including information about abnormal gene expression, somatic mutations, epigenetic modifications, and clinical data among others. In total, 25,000 tumor genomes are compiled in the ICGC. The corresponding clinicopathological characteristics, such as gender, age, stage, etc., and prognostic information of TCGA-COAD patients were downloaded from the UCSC Xena website (http://xena.ucsc.edu/). RNA sequencing data (count value) of 399 samples (TCGA-COAD) with corresponding mutation and survival data were obtained from TCGA database for subsequent analysis. The GRCh38 version of the genome in the Ensembl database (ftp://ftp.ensembl.org/pub/current_gtf) was used for annotation (Howe et al., 2021). In addition, copy number variation (CNV) data were downloaded from TCGA database. The clinical characteristics of the patients are shown in Supplementary Table S1.

### Mutation Analysis

With the development of tumor genomics, the mutation annotation format (MAF) is being widely accepted and used to store detected somatic mutations. In this study, the maftools package (Mayakonda et al., 2018) and the GenVisR package (Skidmore et al., 2016) were used to visualize the somatic mutation data downloaded from TCGA. The somatic mutation data of COAD patients from the ICGC were visualized using the GenVisR package. The G3viz package (Guo et al., 2020) was used to visualize the *FREM2* mutations. In addition, to check whether the CNVs of this gene were associated with COAD, GISTIC2.0 of the Genepattern (https://cloud.genepattern.org/) cloud analysis platform was used to analyze the CNV data obtained from TCGA database (Reich et al., 2006).

### Analysis of the Effects of *FREM2* Mutations on the Prognosis of Patients With COAD

According to the gene expression data of COAD patients downloaded from TCGA, the patients were divided into mutation group (*n* = 36) and wild type group (*n* = 278) according to the *FREM2* mutation status. Survival analysis was performed to study the prognostic difference between the mutation and the wild type groups based on the information about the prognosis of patients with COAD. Additionally, all patients with COAD, whose gene expression data was available, were randomly divided into training (*n* = 266) and test (*n* = 90) sets at a ratio of 3:1. A robust model of *FREM2* mutation prediction was constructed on the training set using the random forest (RF) method (Yperman et al., 2020). The performance of the model was evaluated using the receiver operating characteristic (ROC) curve.

### Construction of the Prognostic Model

The gene expression data of 36 *FREM2*-mutant COAD patients with clinical information were used to construct a prognostic model. First, a univariate Cox regression analysis was performed to initially identify the genes related to OS (*p* value <0.05). Next, a prognostic risk model was established using the RF method and multivariate Cox regression analysis. The risk score calculation formula was:Risk score = exp gene 1 * β gene 1 + exp gene 2 * β gene 2 + exp gene 3 * β gene 3 +… exp gene n * β gene n (exp gene n: the expression level of gene n; β gene n: the regression coefficient of the multivariate Cox regression analysis of gene n). Next, correlation analysis of *FREM2* mRNA expression levels with risk scores and the expression levels of characteristic genes in the model were conducted.

### Evaluation of the Efficacy and Clinical Relevance of the Prognostic Models

According to the median risk score, *FREM2*-mutant COAD patients with clinical information were divided into high-risk and low-risk groups. Kaplan–Meier (K–M) survival curve analysis and time-dependent ROC were used to analyze overall survival (OS) to evaluate the prediction accuracy of the model. Next, among COAD patients with *FREM2* mutations, univariate and multivariate Cox regression analyses were performed using clinicopathological variants*,* such as age, gender, clinical stage, and tumor stage, as well as risk score of patients. Lastly, the correlation between the risk score and clinical characteristics was analyzed.

### Analysis of TMB and MSI

Considering that different types of *FREM2* mutations may have different roles in tumorigenesis, the expression data of COAD patients were divided into inactivating mutation subgroups and other non-silent mutation subgroups. K–M survival curve and time-dependent ROC were used to analyze the prognosis of the two subgroups.

TMB refers to the total number of somatic mutations in the exon coding region of the genome that have substitutions, insertions, or deletions per Mb base in a tumor sample. The TMB score of each sample depicts the total number of somatic mutations (including non-synonymous point mutations, insertions, and deletions in the exon coding region)/target area size, and the unit is mutations/Mb (Chan et al., 2019). A microsatellite is segment of tandem repeats in the human genome, such as single nucleotide repetitions or dinucleotide repetitions. MSI refers to the change of any length of microsatellite caused by the insertion or deletion of repeat units in tumor tissues compared to normal tissues (Hile et al., 2013). MSI is calculated as the number of insertions or deletions in gene repeats. In his study, we separately analyzed the relationship between the risk score of the prognosis model with TMB and MSI.

### Identification of Differentially Expressed Genes

To investigate the effects of *FREM2* mutation on the gene expression levels, samples in TCGA data set were divided into *FREM2*-mutant type and *FREM2*-wild type according to their mutation status. Then, the R package limma was used to analyze the differences between the groups (Ritchie et al., 2015). The thresholds for considering a gene as differentially expressed were set as |log fold change (logFC)| > 0.5 and *p* value < 0.05. Genes with logFC >0.5 and *p* value <0.05 were considered to be differentially up-regulated and those with logFC < −0.5 and *p* value < 0.05 were considered to be differentially down-regulated. The results of this analysis were displayed using heat map and volcano plot.

### Gene Function and Pathway Enrichment Analysis

Gene Ontology (GO) enrichment analysis is a common method for large-scale functional enrichment studies of genes in different dimensions and at different levels, generally from three levels: biological process, molecular function, and cellular component (Ashburner et al., 2000). Kyoto encyclopedia of genes and genomes (KEGG) (Kanehisa and Goto, 2000) is a widely used database that contains information about genomes, biological pathways, diseases, and drugs. We used the R software package clusterProfiler (Yu et al., 2012) to perform GO function annotation and KEGG biological pathway enrichment analysis on differentially expressed genes to identify significantly enriched biological processes and pathways. *p* value <0.05 was considered statistically significant.

### Gene Set Enrichment Analysis (GSEA) and Gene Set Variation Analysis (GSVA)

GSEA is a n method used to determine whether a set of predefined genes show statistical differences between two biological states. It is generally used to estimate changes in pathway and biological process activity in expression data sets (Subramanian et al., 2005). In order to study the differences in the biological processes of genes between the *FREM2*-mutant and the *FREM2*-wild type groups, the reference gene sets “C5.go.v7.4.symbols.gmt” and “c2.cp.kegg.v7.4. symbols. gmt” were downloaded from the MSigDB database (Liberzon et al., 2015). The R package “clusterProfiler” was used to perform GSEA on TCGA-COAD gene expression profile data. *p* value <0.05 was considered statistically significant.

GSVA (Liberzon et al., 2015) is a non-parametric unsupervised analysis method that relies on converting the expression matrix of genes between different samples into the expression matrix of gene sets between samples to evaluate the gene set enrichment results of the transcriptome, and to further evaluate whether different metabolic pathways are enriched in different samples. In order to study the biological process that were altered in the *FREM2-*mutant group compared to the *FREM2*-wild type group, GSVA was performed using the R package “GSVA” (Hanzelmann et al., 2013). The reference gene set “h.all.v7.4.symbols.gmt” from the MSigDB database was downloaded to calculate the enrichment score of each sample in the data set in each pathway. Finally, the correlation between the GSVA results and the risk score was analyzed.

### Immune-Cell Infiltration Analysis

The immune microenvironment is a complex integrated system mainly composed of immune cells, inflammatory cells, fibroblasts, interstitial tissues, and various cytokines and chemokines. Analysis of immune cell infiltration in tissues is an important tool in understanding the pathological mechanisms of a disease and guiding prognosis prediction.

ESTIMATE is an algorithm that quantifies the immune infiltration level in tumor samples based on gene expression data, which can reflect the diversity of the stroma and immune cells. In this study, the estimate package in R (Yoshihara et al., 2013) was used to estimate the content of stromal cells and immune cells in TCGA-COAD. The correlation between the characteristic genes of the prognosis model and the expression levels of *FREM2* and the ESTIMATE score were analyzed.

CIBERSORT is an algorithm that deconvolves the expression matrix of immune cell subtypes based on the principle of linear support vector regression using RNA-Seq data to estimate the abundance of immune cells in the tissue. In this study, the proportion of 22 immune cell subtypes in TCGA-COAD immune microenvironment was evaluated using the CIBERSORT algorithm (Newman et al., 2019) in R software. The number of permutations was set to 1,000, and a *p* value <0.05 was considered be representative of an accurate sample for calculating the content of immune cells. Using Pearson correlation analysis, the correlation between the expression of characteristic genes of the prognostic model and the expression levels of *FREM2* and 22 types of immune cells in COAD was calculated.

To examine the biological processes and cell signaling pathways that the characteristic genes of the prognostic model may participate in, the immune gene set from the ImmPort database (Bhattacharya et al., 2014) (https://www.immport.org) was downloaded and the relationships between characteristics genes of the prognostic model and *FREM2* and the immune genes were analyzed. Major histocompatibility complex (MHC) is expressed on the cell surface of all nucleated cells, and the human MHC is collectively referred to as human leukocyte antigen (HLA). HLA is a key molecule in antigen presentation and antigen recognition by immune cells. The relationships between the expression levels of members of the HLA family and the risk score of the prognostic model was also analyzed.

### Patients Tissue Specimens

A total of 30 patients fulfilling the inclusion criteria (histologically confirmed stage II or III or IV melanoma) at The First People’s Hospital of Foshan between 2019 and 2021 were included in the present study (Supplementary Table S2). The exclusion criteria were as follows: *1*) Incomplete previous medical history, immunohistochemistry (IHC) information, and follow-up information; *2*) cancer recurrence post-surgery; *3*) patients with multiple tumors; *4*) patients who received radiotherapy/chemotherapy before surgery. Patient-informed consent was obtained and approved by The First People’s Hospital of Foshan Subject Review Board.

### IHC Staining and Analysis

IHC staining was performed as previously described elsewhere (Yang et al., 2021). Briefly, specimens were incubated with individual primary antibodies (anti-FREM2, 1:50, Atlas Antibodies; anti-Ki-67,1:100, Abcam) and then washed and incubated with horseradish peroxidase–conjugated secondary antibody (goat anti-rabbit, 1:500, Cell Signaling Technology). Colorimetric reaction was using diaminobenzidine (DAB).

All specimens were examined using the cross-product (H score) of the percentage of tumor cell staining at each of the three staining intensities. The intensity of immunopositivity was scored as follows: none, 0; weak, 1; moderate, 2; and strong, 3. For example, a particular tumor may have 50% cell staining at intensity = 1 and 50% of cell staining at intensity = 3, it would have a combined H score of 200 [(50 × 1) + (50 × 3) = 200], with a range from 0 to 300. The final score was graded by H score as follows: Low, H score 0–100; Moderate, H score 101–200; and High, H score 201–300. All IHC sections were scored blindly by three independent pathologists. The IHC score were agreed upon by at least two out of three pathologists.

### Expression Levels of *FREM2* in Pan-Cancer and COAD

UALCAN (http://ualcan.path.uab.edu/index.html) is an effective online analysis and mining website for cancer data, mainly based on the relevant cancer data in TCGA database (Chandrashekar et al., 2017). UALCAN database was used to analyze the expression levels of *FREM2* in pan-cancer and COAD. The Human Protein Atlas (HPA, https://www.proteinatlas.org/) is a comprehensive database that provides the protein expression profiles for a large number of human proteins, presented as immunohistological images from most human tissues. The HPA database was used to detect the expression of FREM2 in COAD tissues.

### Statistical Analysis

All data calculation and statistical analysis were performed using R (https://www.r-project.org/, version 4.1.0). Benjamini-Hochberg was used for multiple test correction, and false discovery rate was used in multiple tests to correct for multiple testing. For the comparison of two groups of continuous variables, normally distributed variables were analyzed using independent Student’s t test, and non-normally distributed variables were analyzed using Mann-Whitney U test (Wilcoxon rank sum test). The survival package of R (Durisova and Dedik, 1993) was used for survival analysis, the K–M survival curve was used to show the difference in survival, and the log-rank test was used to evaluate the significance of the difference in survival time between the two groups. Univariate and multivariate Cox analyses were used to determine independent prognostic factors. pROC and ROCR packages were used to construct the ROC curve (Sing et al., 2005; Robin et al., 2011), and the area under the curve (AUC) was used to evaluate the accuracy of prognosis estimated by the risk score. All *p* values were two-sided, and *p* value <0.05 was considered statistically significant.

## Results

### Identification of the *FREM2* Mutation Frequency in COAD

**FIGURE 1:**
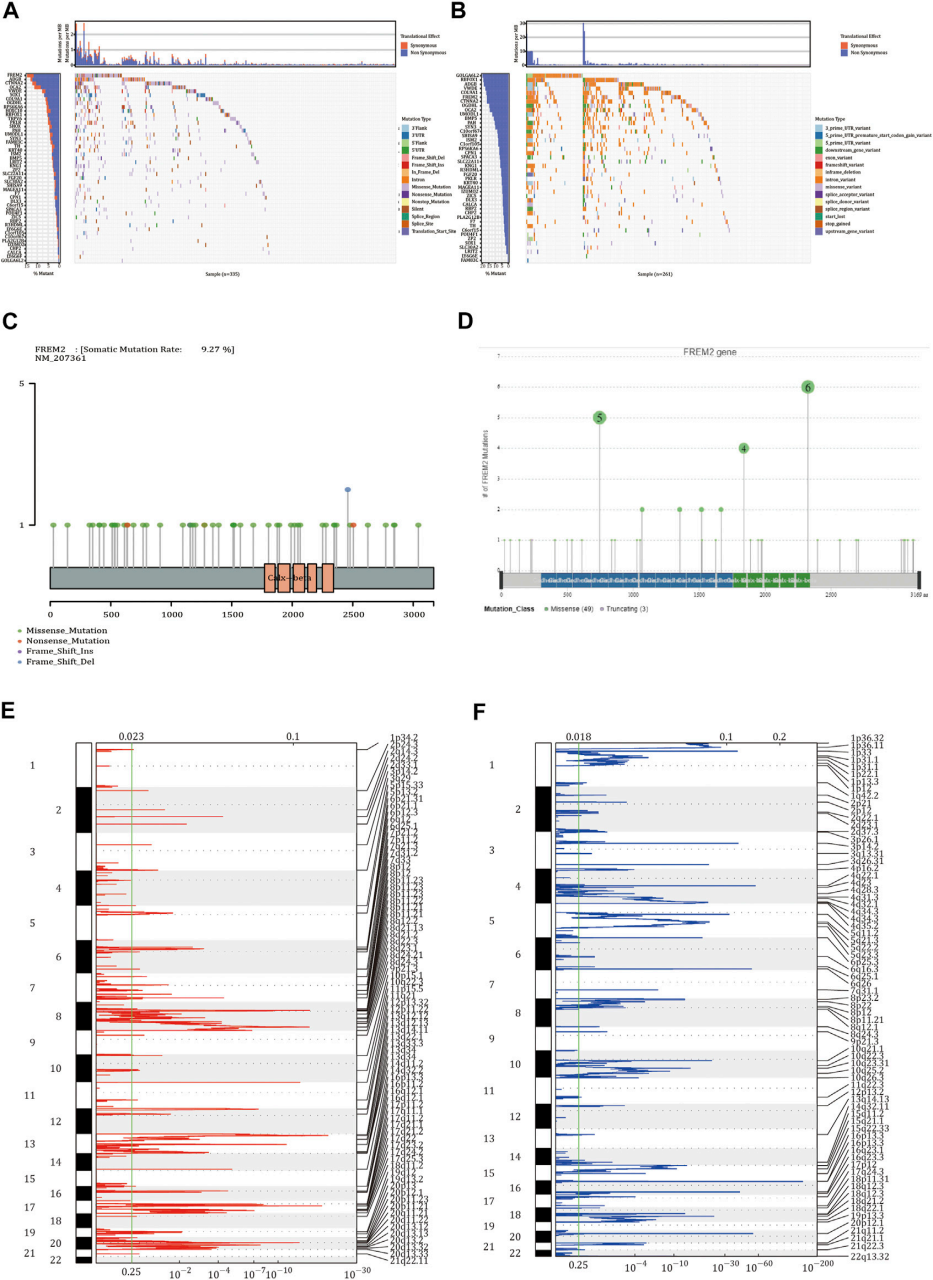
Somatic mutation and copy number variation analysis in colon adenocarcinoma (COAD) patients. **(A)** The 68 genes with the highest mutation frequency in COAD patients from The Cancer Genome Atlas (TCGA). **(B)** The mutations of 68 genes in the International Cancer Genome Consortium (ICGC) database. In the two waterfall charts, the left panel shown genes with high frequency mutations arranged according to their mutation frequency and the right panel shows different types of mutations represented by various color modules. **(C)** The mutations of *FREM2* in TCGA cohort and **(D)** in the ICGC cohort. **(E**,**F)** Identification the amplification and deletion of *FREM2*. The mRNA located at the focal copy number alteration peak was related to COAD. The false discovery rate (Q value) and the change score of GISTIC2.0 (*x*-axis) correspond to the position of the genome (*y*-axis). The dotted line indicates the centromere. The green line represents the 0.25 Q value cutoff point for determining significance.

We identified 68 genes with somatic mutation data in TCGA-COAD patients obtained from TCGA (Figure 1A). Additionally, these 68 genes were also identified in the data downloaded from the ICGC database (Figure 1B). As shown in Figures 1C,D, the mutation frequency of *FREM2* was relatively high, and the mutation of FREM2 was visualized. We used GISTIC 2.0 to identify genes that exhibited significant amplification or deletion using the CNV data in TCGA. *FREM2* did not show significant amplification or deletion (Figures 1E,F).

### Construction of *FREM2* Mutation Prediction Model

**FIGURE 2:**
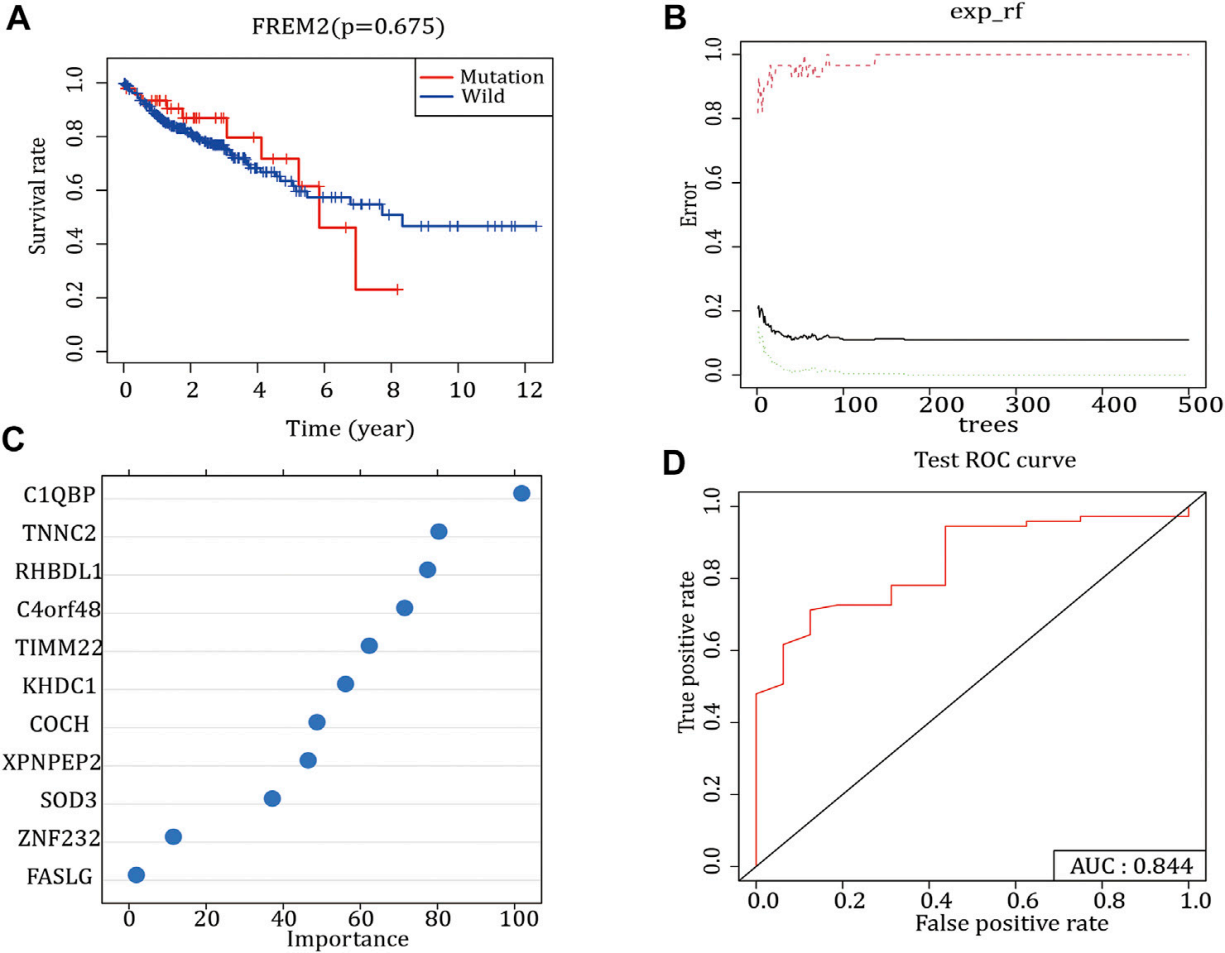
*FREM2* mutation survival analysis and model construction. **(A)** The effect of *FREM2* mutation on the overall survival time of the patients. Blue indicates *FREM2*-wild type, and red indicates *FREM2-*mutant type. **(B**,**C)** Random Forest method to construct *FREM2* mutation model. **(D)** Performance of the *FREM2* mutation model in the test set.

Survival analysis was performed according to the *FREM2* mutation and prognostic information of patients with COAD. The results showed that *FREM2* mutations significantly impacted the prognosis and survival of patients with COAD (Figure 2A). In the training set, the RF method was used to construct a *FREM2* mutation prediction model based on the mRNA data (Figures 2B,C). The ROC curve and the AUC were used to evaluate the performance of the model. An AUC value close to 1 indicates that the model has a high sensitivity at a very low false-positive rate. The AUC value of the model in the training cohort was 1.00, and the AUC value in the validation cohort was 84.4% (Figure 2D), indicating that the performance of the model was sufficient to effectively predict *FREM2* mutations in other cohorts.

### Construction of a Prognostic Model

**FIGURE 3:**
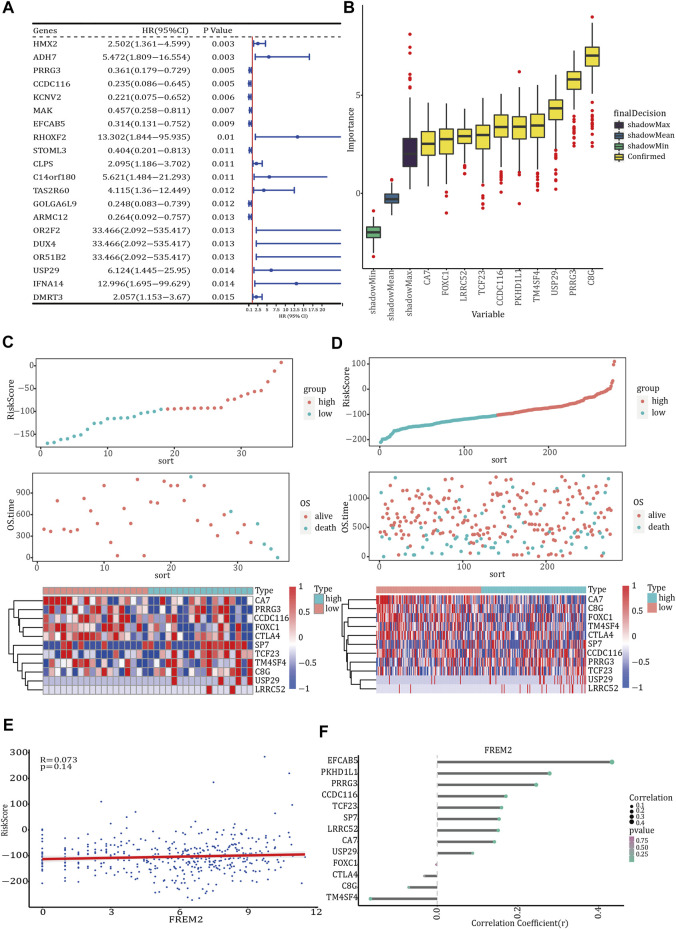
Prognosis model of *FREM2* mutation. **(A)** The forest plot of the top 20 prognosis-related genes obtained by univariate Cox regression analysis. The left side of the vertical dashed line shows the protective gene, and the right-side shows the risk gene. **(B)** The 13 important features selected based on random forest. Risk score, survival status and characteristic gene expression analysis of *FREM2*-mutant type **(C)** and *FREM2*-wild type **(D)**. **(E)** Scatter plot of the correlation between *FREM2* expression and risk score. **(F)** Correlation between the expression levels of *FREM2* and characteristic genes. The size of the dot represents the strength of the correlation between *FREM2* and the characteristic gene; the larger the dot, the stronger the correlation, and vice versa. The color of the point represents the *p* value. The greener the color, the smaller the *p* value, and the pinker the color, the larger the *p* value. *p* value <0.05 was considered statistically significant.

Using the gene expression data of 36 *FREM*-mutant COAD patients with clinical information, univariate Cox regression analysis was performed to initially identify 20 genes related to OS (*p*-value <0.05) (Figure 3A). Next, we used the RF method to select the most important genes related to prognosis. The results identified a total of 13 genes: *FOXC1, PRRG3, USP29, CCDC116, LRRC52, CTLA4, TCF23, CA7, TM4SF4, SP7, C8G, EFCAB5*, and *PKHD1L1* (Figure 3B). Next, multivariate Cox regression analysis clarified the correlation between these 13 genes and OS. The Cox regression coefficients of the 13 characteristic genes were calculated and used to estimate the risk score of each sample, which was calculated as the sum of the expression levels of each characteristic gene multiplied by their regression coefficients.

We evaluated the predictive performance of the prognostic model using the *FREM2*-mutant and *FREM2-*wild type groups. Based on the prognostic model, the risk scores of COAD patients were calculated and sorted, and the survival status of each patient was displayed on a dot plot (Figures 3C,D). The correlation between *FREM2* expression levels and risk score and characteristic genes expression levels was analyzed. The expression level of *FREM2* was positively correlated with the risk score (Figure 3E). Additionally, the expression level of *FREM2* was significantly positively correlated with that of *PRRG3* (*r* = 13.651), *USP29* (*r* = 56.206), CC*DC116* (*r* = 11.403), *LRRC52* (*r* = 44.466), TCF*23* (*r* = 9.083, *TM4SF4* (*r* = 0.003), *SP7* (*r* = 8.531), and *EFCAB5* (*r* = 5.282), and negatively correlated with that of *FOXC1* (*r* = −10.22), *CTLA4* (*r* = −5.152), *CA7* (*r* = −11.705), C8G (*r* = −2.951), and *PKHD1L1* (*r* = −20.17) (Figure 3F).

### Evaluation of the Prognostic Model

**FIGURE 4:**
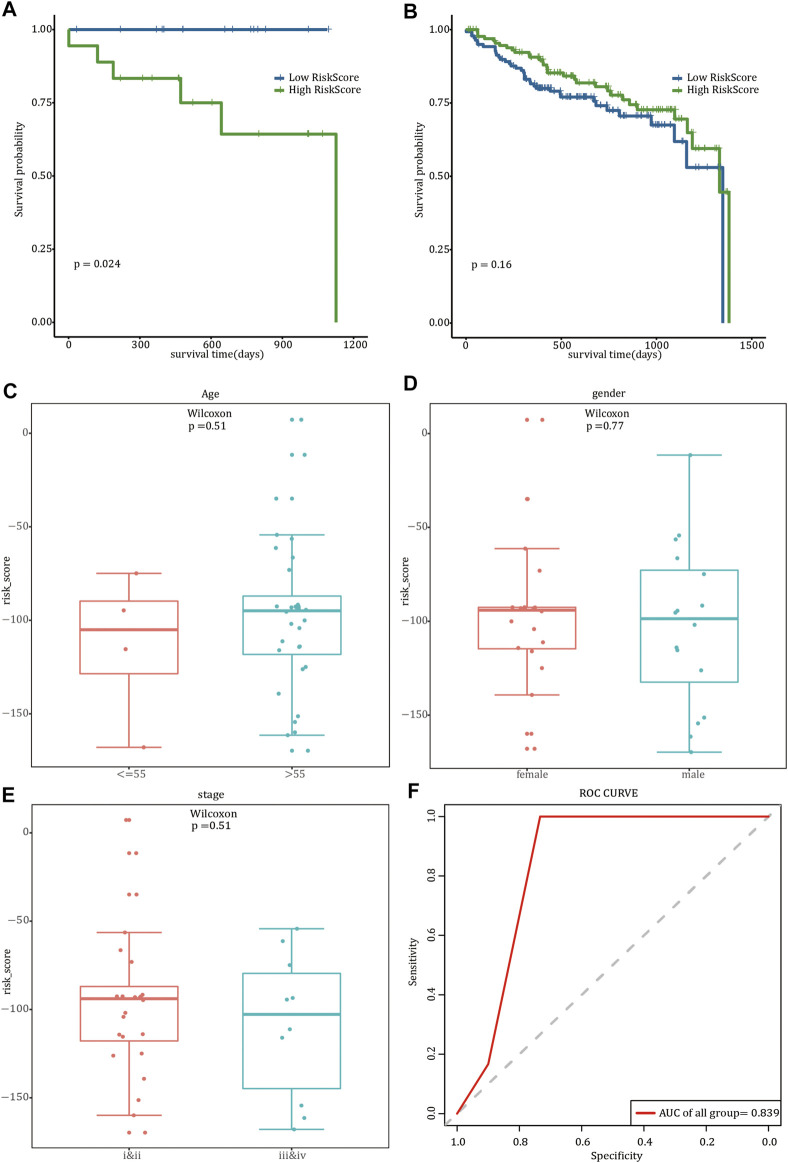
Prognostic model analysis and clinical model construction. The effect of risk score on overall survival of *FREM2*-mutant type **(A)** and *FREM2*-wild type **(B)** patients. Blue indicates low risk score, and green indicates high risk score. Correlation analysis of risk score with age **(C)**, gender **(D)**, and tumor stage **(E)**. **(F)** The receiver operating characteristic curve of the clinical prediction model in 36 *FREM2*-mutant samples.

According to the median risk score, *FREM2*-mutant COAD patients with clinical information were divided into high-risk and low-risk groups. The results of survival analysis showed that there was a significant difference in OS between the two risk groups in which the 36 *FREM2*-mutant samples had been divided (Figure 4A). However, there was no significant difference in OS between the high- and low-risk groups in which the 278 *FREM2*-wild type samples were divided (Figure 4B). The correlation analysis between the risk score and the clinical characteristics of the 36 FREM2-mutant samples showed that there were no significant differences in risk scores across different ages, genders, and tumor stages (Figures 4C–E). According to the age, gender, tumor stage, and risk score of COAD patients with *FREM2* mutations, univariate Cox analysis and multivariate Cox analysis were performed to construct a clinical prediction model. The efficacy of the model in 36 FREM2-mutant samples was 83.9% (Figure 4F).

### Analysis of TMB and MSI

Considering that different *FREM2* mutation types may have different roles in the occurrence of rectal cancer, we divided the 36 *FREM2*-mutant COAD patients into two subgroups: patients with inactivating mutations (*n* = 27, including non-sense mutations and silent mutations), and patients with other non-silent mutation (*n* = 55).

**FIGURE 5:**
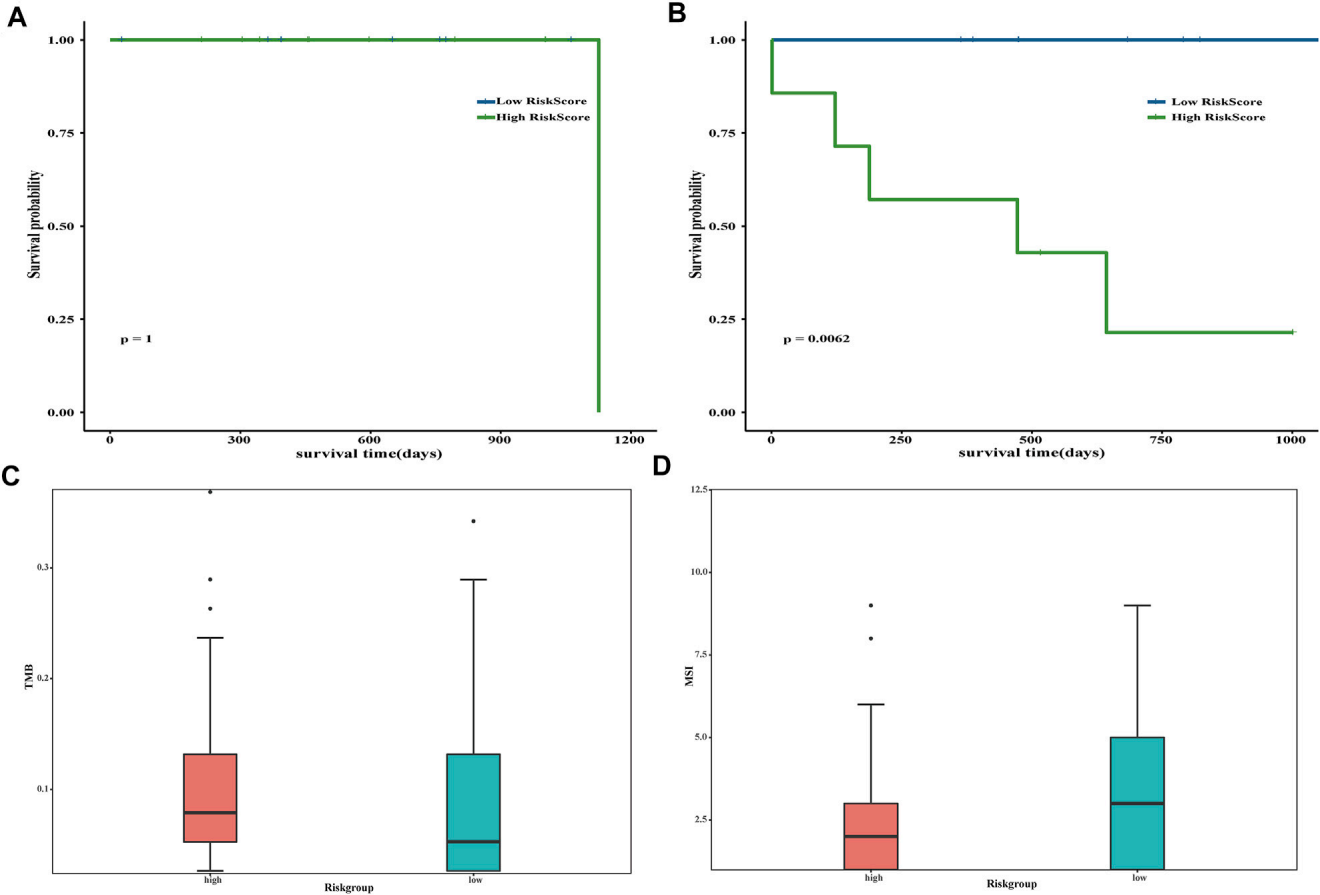
Evaluation of risk score. The impact of risk scores on overall survival in the subgroup of inactivating mutations **(A)** and other non-silent mutations subgroups **(B)**. Blue indicates low-risk scores, and green indicates high-risk scores. Correlation analysis between tumor mutation burden **(C)** and microsatellite instability **(D)** and risk scores. Pink is used to represent the group with high risk, whereas green represents the group with low risk.

The prognosis of the low-risk group was significantly better than that of the high-risk group, and limited by the insufficient sample size, we only performed a 1-year time-dependent ROC analysis (Figures 5A,B). We obtained TMB scores based on the total number of mutations and calculated the relationship between TMB and the risk scores. Significant differences were shown in TMB between samples with different risk scores (*p* value <0.05) (Figure 5C). Next, the risk score and MSI were analyzed, and there were also significant differences in MSI between samples with different risk scores (*p* value <0.05) (Figure 5D).

### Identification of Differentially Expressed Gene and Functional Enrichment Analysis

**FIGURE 6:**
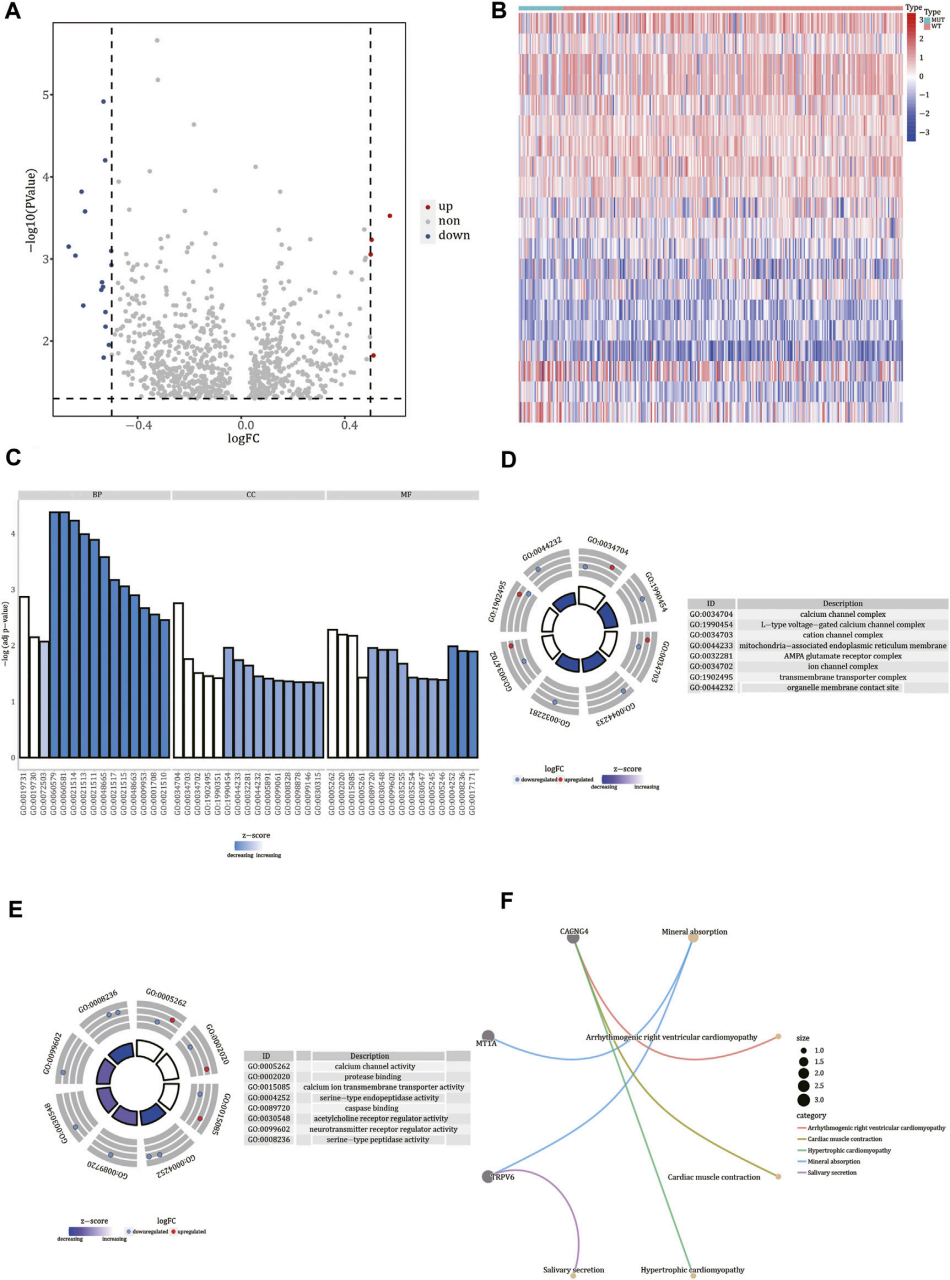
Functional enrichment analysis of differentially expressed genes. **(A)** Volcano plot of differentially expressed genes. The red nodes indicate up-regulation, blue nodes indicate down-regulation, and gray nodes indicate non-significant expression changes. **(B)** Heat map of differentially expressed genes. Red represents high gene expression levels, blue represents low gene expression levels, green annotation bars indicate *FREM2-*mutant samples, and red annotation bars indicate *FREM2*-wild type samples. The result of Gene Ontology functional enrichment analysis of differentially expressed genes **(C)**, and the results of molecular function **(D)** and cell compartment **(E)** terms enrichment analysis are displayed. Blue indicates down-regulation of expression, red indicates up-regulation of expression, the middle quadrilateral indicates the effect of the gene on the enriched Gene Ontology terms, light color indicates inhibition, and dark color indicates activation. **(F)** The top five pathways of Kyoto Encyclopedia of Genes and Genomes enrichment analysis of differentially expressed genes.

To identify the differentially expressed genes in *FREM2*-mutant and *FREM2-*wild-type samples, we used the limma R package. Based on the gene expression profile data of 36 *FREM2*-mutant samples and 278 *FREM2*-wild type samples in TCGA-COAD, we found four up-regulated genes (*p* value <0.05, logFC > 0.5) and 16 down-regulated genes (*p* value <0.05, logFC < −0.5). Differentially expressed genes were visualized using a volcano plot and a heat map (Figures 6A,B).

To determine the functions of the differentially expressed genes, we analyzed the biological processes, cell components, and molecular functions in which they were involved according to GO enrichment analysis (Figure 6C and Supplementary Table S3). GO analysis results showed that the 20 differentially expressed genes were significantly enriched in calcium channel complex, L-type voltage-gated calcium channel complex, cation channel complex, mitochondria-associated endoplasmic reticulum membrane, AMPA glutamate receptor complex, ion channel complex, transmembrane transporter complex, organelle membrane contact site, and other cellular components (Figure 6D). Additionally, these genes were involved in molecular functions, such as calcium channel activity, protein binding, calcium ion transmembrane transporter activity, serine-type endopeptidase activity, caspase binding, acetylcholine receptor regulator activity, serine-type peptidase activity, and neurotransmitter receptor regulator activity (Figure 6E). Finally, using KEGG enrichment analysis, we also analyzed the pathways in which the 20 differentially expressed genes were involved (Supplementary Table S4). According to the results, these genes were involved in pathways such as mineral absorption, salivary secretion, cardiac muscle contraction, and hypertrophic cardiomyopathy (Figure 6F).

### GSEA and GSVA

**FIGURE 7:**
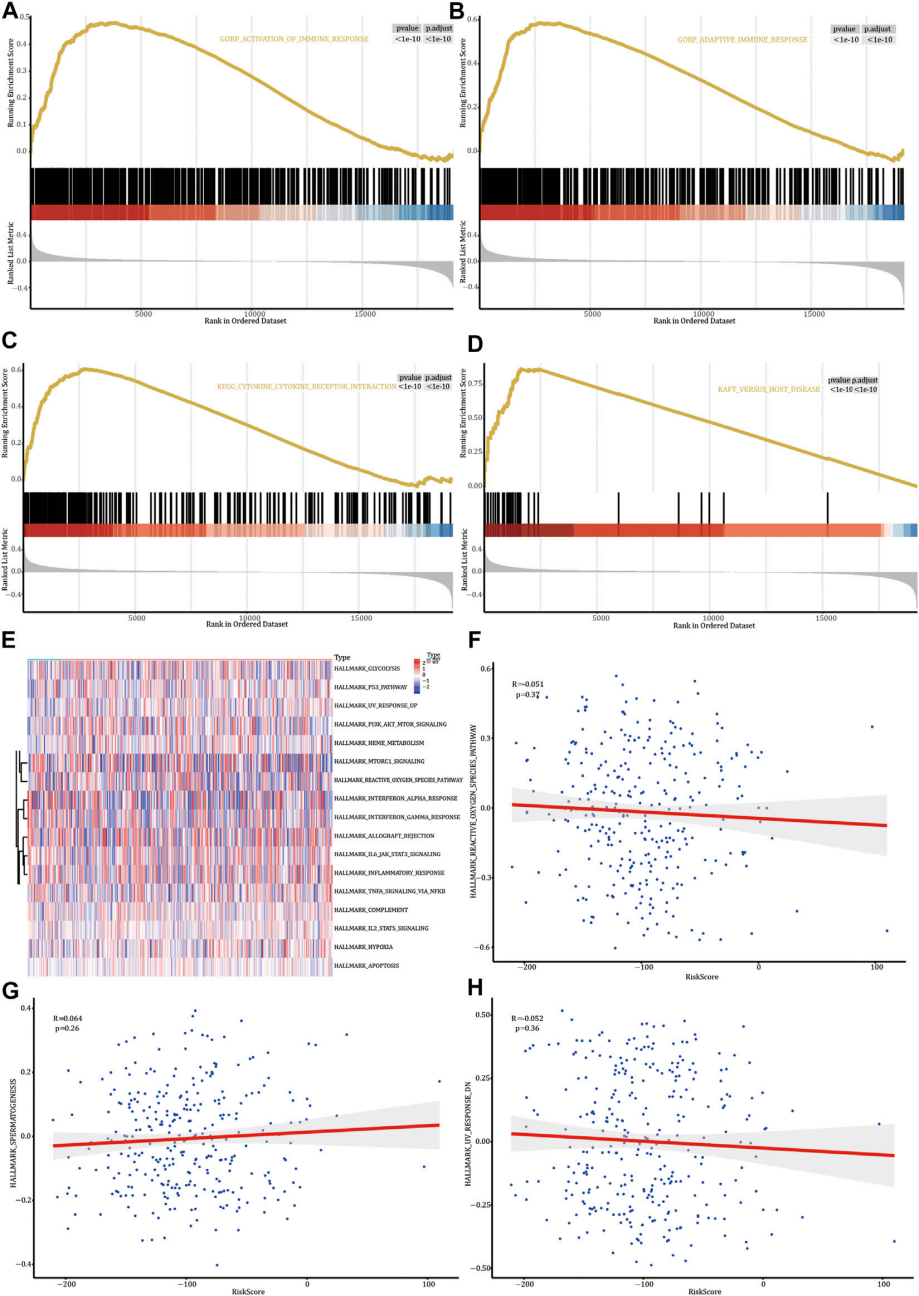
Gene Set Enrichment Analysis (GSEA) and Gene Set Variation Analysis (GSVA). GSEA biological function enrichment analysis shows activation of the immune response **(A)** and adaptive immune response **(B).** GSEA biological pathway enrichment analysis results show cytokine-cytokine receptor interaction **(C)** and adaptive immune response **(D)**. **(E)** Heat map of significant hallmarks analyzed using GSVA. Scatter plot of correlation between significant hallmark and risk score: reactive_oxygen_species_pathway **(F)**, spermatogenesis **(G)**, and uv_response_dn **(H)**.

GSEA on the genes differentially expressed in *FREM2*-mutated and *FREM2-*wild type patients showed that the genes were significantly enriched in biological functions, such as the activation of immune response and adaptive immune response (Figures 7A,B and Supplementary Table S5), and enriched in pathways such as the cytokine-cytokine receptor interaction and graft versus host disease (Figures 7C,D and Supplementary Table S5).

Next, we analyzed the genes differentially expressed in *FREM2*-mutated and *FREM2-*wild type patients to analyze the role of these genes using GSVA. The results showed that 17 hallmark pathways were differentially enriched in *FREM2*-mutated and *FREM2-*wild type patients (Figure 7E). Among them, spermatogenesis was positively correlated with risk score, while reactive_oxygen_species_pathway and uv_response_dn were negatively correlated with risk score. Other correlations were not significant (*p* value < 0.05) (Figures 7F–H).

### Immune Cell Infiltration Analysis

**FIGURE 8:**
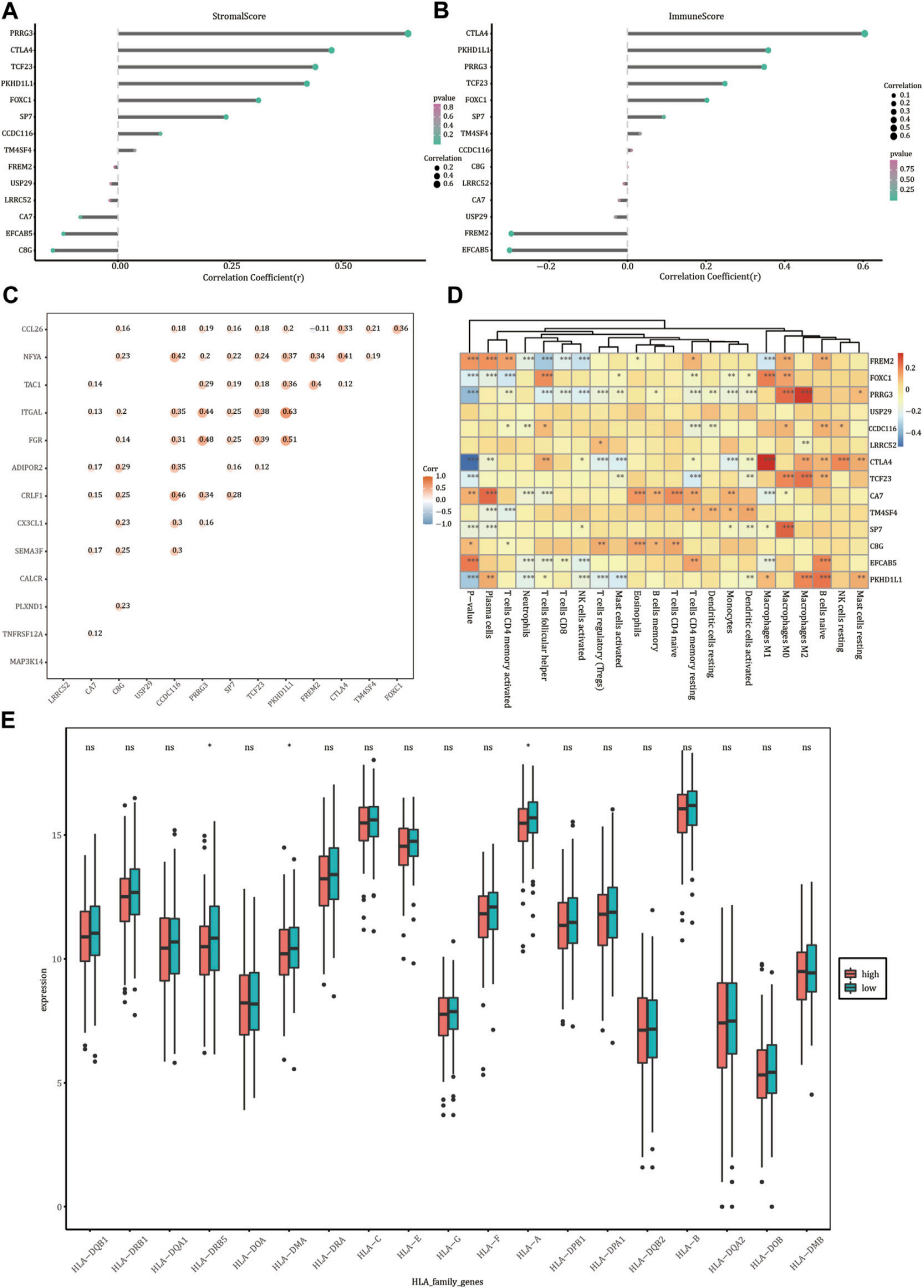
Immune correlation analysis. Correlation of the expression levels of *FREM2* and characteristic genes with the stromal cells **(A)** and the abundance of immune cells **(B)**. **(C)** Correlation between *FREM2* and characteristic genes and immune genes. **(D)** Correlation between *FREM2* and characteristic gene expression levels and immune cell infiltration. **(E)** Correlation between members of the *HLA* family expression levels and risk score.

We analyzed the relationship between the expression levels of *FREM2, FOXC1, PRRG3, USP29, CCDC116, LRRC52, CTLA4, TCF23, CA7, TM4SF4, SP7, C8G, EFCAB5,* and *PKHD1L1* and the abundance of immune cells and stromal cells (Figures 8A,B). Stromal cell abundance was significantly positively correlated with the expression levels of *PRRG3, CTLA4, TCF23, PKHD1L1, FOXC1,* and *SP7*, and significantly negatively correlated with the expression levels of *EFCAB5* and *C8G*. The abundance of immune cell types was significantly positively correlated with the expression levels of *FOXC1, PRRG3, CTLA4, TCF23,* and *PKHD1L1*, and significantly negatively correlated with the expression levels of *FREM2* and *EFCAB5* (*p* < 0.05). *FREM2* expression levels were significantly related with the expression levels of immune genes such as *TAC1, NFYA,* and *CCL26*; *PKHD1L1* was significantly related with the expression levels of the immune genes *ITGAL* and *NFYA*; *FOXC1* was significantly related with the expression levels of the immune gene *CCL26* (*p* value < 0.05) (Figure 8C). *FREM2* and *PKHD1L1* gene expression levels were significantly correlated with the infiltration rate of 12 types of immune cells; *FOXC1* gene expression levels were significantly correlated with the infiltration rate of 10 immune cells (*p* value < 0.05) (Figure 8D). The expression value of *HLA-DOA* differed in the two different risk groups (Figure 8E).

### FREM2 Protein Level Analysis

**FIGURE 9:**
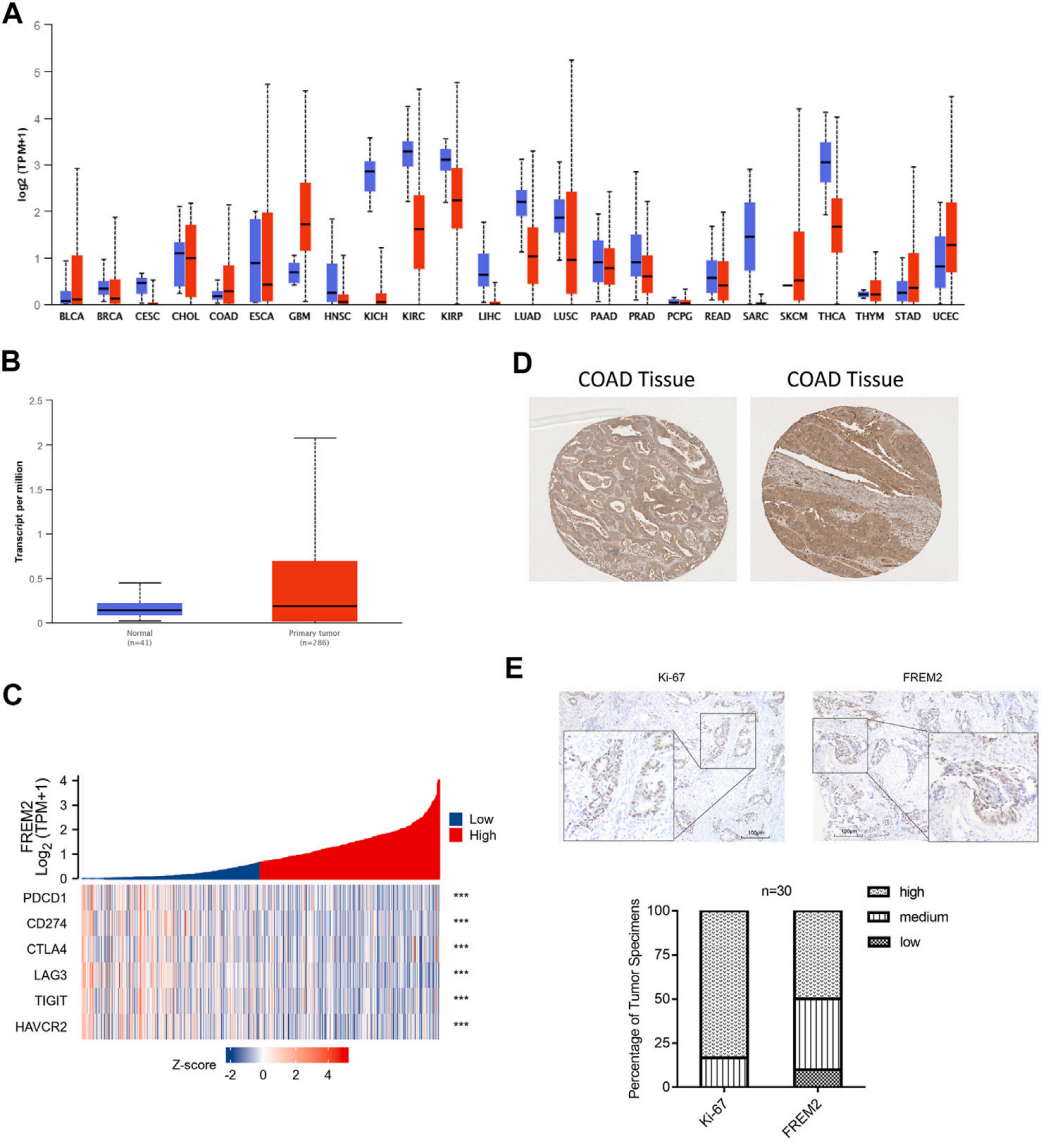
*FREM2* expression analysis. Expression of *FREM2* in pan-cancer, using UALCAN database **(A)**. The expression of *FREM2* in colon adenocarcinoma (COAD), using the data from The Cancer Genome Atlas database **(B)**. Correlation analysis of *FREM2* and immune checkpoints, displayed using a heat map **(C)**. Analysis of FREM2 expression levels in COAD tissues, using the Human Protein Atlas (HPA) database **(D)**. High expression levels of Ki-67 and FREM2 were presented in colorectal cancer tissues (*n* = 30) by immunohistochemistry (IHC) staining. **(E)** Histogram of the results of analysis of IHC staining. Original magnification is ×100 (inset: IHC stain, DAB, original magnification is ×400).

We used the UALCAN database to analyze the expression levels of FREM2 in pan-cancer and found that FREM2 was mainly highly expressed in COAD, glioblastoma multiforme (GBM), stomach adenocarcinoma (STAD), and uterine corpus endometrial carcinoma (UCEC) (Figure 9A). Further analysis of COAD tissue samples showed that FREM2 was highly expressed in tumor tissues compared to normal tissues (Figure 9B). In addition, the expression levels of FREM2 in COAD tissues was analyzed using the HPA database, and it was found that FREM2 was highly expressed in tumor tissues (Figure 9D). Next, we evaluated FREM2 and Ki-67 expression levels in 30 CRC tissues using histochemistry staining. As shown in Figure 9E, histological scoring and analysis revealed that FREM2 and Ki-67 were highly expressed in tissue specimens from CRC patients, which was consistent with the results of the previous analysis. Finally, we examined the role of FREM2 molecular function. PDCD1, CD274, CTLA4, LAG3, TIGIT, and HAVCR2 are important immune checkpoints responsible for tumor immune escape. Given the regulatory role of FREM2 in COAD, the relationship of FREM2 to PDCD1, CD274, CTLA4, LAG3, TIGIT, and HAVCR2 was assessed. As shown in Figure 9C, FREM2 expression was significantly correlated with that of PDCD1, CD274, CTLA4, LAG3, TIGIT, and HAVCR2. These results suggested that FREM2 was highly expressed in COAD and that tumor immune escape may be involved in FREM2-mediated COAD carcinogenesis.

## Discussion

With the wide application of endoscopy technology and the yearly increase in the number of physical examinations, more and more patients with colon cancer are detected early, which increases the chances of a favorable outcome after surgery. Although with the maturity of laparoscopic surgery technology and the development of neoadjuvant chemotherapy have contributed to improve the survival rate of CRC patients after surgery, the 5-year survival rate is still less than 65%. Therefore, it is necessary to identify new prognostic biomarkers in CRC patients. The occurrence of CRC is a multi-step process, including chromosomal abnormalities, gene mutations, and epigenetic changes. These abnormalities may be associated with patient survival. For example, while *KRAS* mutations generally occur relatively early in the evolution of CRC, mainly during the transformation of small to neutral adenomas, mutations in TP53 often occur in later stages. Additionally, previous studies have shown that the number of somatic mutations is positively correlated with the response to immunotherapy (Link and Overman, 2016).

*FREM2* is located at 13q13.3 and forms an independent and complete ternary complex structure (FREM2-FRAS1-FREM1) between the extracellular epithelium and the mesenchyme (Kantaputra et al., 2021). The functions of this complex are similar to those of Collagen VII, and each component of the complex is essential to maintain the stability of the complex structure (Dalezios et al., 2007).

In humans, *FREM2* gene mutations can cause Fraser syndrome, a rare autosomal recessive genetic disease (Jadeja et al., 2005). Additionally, recent studies have shown that *FREM2* mutations cause metabolic reprogramming of mouse embryos during cryptographic development (Zhang et al., 2020), and that loss of function mutations of *FREM2* can disrupt the morphogenesis of the eye (Zhang et al., 2019). Additionally, loss of FREM2 function is an important cause of blood-related kidneys (Al-Hamed et al., 2021), and FREM2 has been suggested to be a candidate prognostic marker in glioma (Vidak et al., 2018).

In this study, we found that *FREM2* had a high mutation frequency in CRC and that *FREM2* mutation was associated with poor prognosis in patients. To further explore the prognostic value of mutations, we divided 36 *FREM2*-mutated patients into high- and a low-risk groups based on the risk scores, constructed a prognostic model, and evaluated its performance. The results suggested that in 36 *FREM2*-mutant patients with CRC, the model showed a higher efficiency, reaching a prediction accuracy of 83.9%. Additionally, we found significant differences in TMB and MSI between the groups with different risk scores. Next, functional enrichment analysis of differentially expressed genes revealed significantly enrichment of genes involved in cytokine-cytokine receptor interaction, immune response, and other pathways. Then, immune infiltration analysis revealed that *FREM2* gene expression was significantly related to the infiltration of 12 immune cell types. Finally, we analyzed the protein expression of FREM2 in pan-cancer and COAD using UALCAN and HPA databases and found that FREM2 was highly expressed in COAD, which was consistent with the results of immunohistochemistry. In addition, since *FREM2* mutation was associated with immune infiltration, we analyzed its association with the expression levels of *PDCD1, CD274, CTLA4, LAG3, TIGIT*, and *HAVCR2,* which are important immune checkpoints responsible for tumor immune escape. FREM2 was significantly correlated with immune checkpoints, which further suggested that FREM2 may regulate immune processes in COAD.

The results of this study should be viewed in light of its limitations. Most of the conclusions were drawn from bioinformatics analysis, and only a small amount of them were validated using clinical samples. In the future, we will continue to further study the functional role of *FREM2* in COAD. Moreover, this study was based on a single omics study, and the understanding of gene function was not comprehensive enough, highlighting the need of more in-depth research in the future. In conclusion, through comprehensive analysis and experimental verification, our results demonstrate that *FREM2* mutations may be prognostic markers for CRC patients.

## Data Availability

Publicly available datasets were analyzed in this study. This data can be found at: http://cancergenome.nih.gov/ and http://xena.ucsc.edu/.

## Abbreviations

AUC, Area under the curve; COAD, colon adenocarcinoma; CRC, colorectal cancer; FPKM, Fragments Per Kilobase per Million; GO, Gene Ontology; GSEA, Gene Set Enrichment Analysis; GSVA, Gene Set Variation Analysis; HLA, human leukocyte antigen; HPA, The Human Protein Atlas; ICGC, International Cancer Genome Consortium; IHC, Immunohistochemistry; KEGG, Kyoto encyclopedia of genes and genomes; K–M, Kaplan-Meier; MHC, Major histocompatibility complex; MSI, microsatellite instability; OS, overall survival; RF, random forest; ROC, receiver operating characteristic; TCGA, The Cancer Genome Atlas; TMB, tumor mutation burden; TPM, transcripts per million reads.
