# Supplementary material for: Identification and Comprehensive Analysis of FREM2 Mutation as a Potential Prognostic Biomarker in Colorectal Cancer
Source: Front Mol Biosci. 2022 Feb 18;9:839617. doi: 10.3389/fmolb.2022.839617 (PMC8896260; doi:10.3389/fmolb.2022.839617)
Supplement: Supplementary file 1 [file DataSheet1.docx]

Supplementary Material

# Supplementary Tables

Supplementary Table 1 Summary of the patient data sets

| **Variable** | **TCGA set (n=571)** | **ICGC set (n=411)** |
| --- | --- | --- |
| Age |  |  |
| <=55 years | 112 | 146 |
| >55 years | 457 | 265 |
| Gender |  |  |
| Female | 271 | 154 |
| Male | 298 | 257 |
| TNM stage |  |  |
| I/II | 316 | 137 |
| III/IV | 241 | 192 |
| Family history |  |  |
| No | N/A | 172 |
| Yes | N/A | 23 |

Supplementary Table 2 Clinical characteristics of patients

| No | Surgery time | sex | age | Distant metastasis | grade | Survival status |
| --- | --- | --- | --- | --- | --- | --- |
| 1 | 2019.08 | M | 47 | No | II | Alive |
| 2 | 2019.07 | F | 56 | No | III | Alive |
| 3 | 2020.02 | M | 75 | No | II | Alive |
| 4 | 2021.01 | M | 65 | No | II | Alive |
| 5 | 2021.01 | M | 66 | No | II | Alive |
| 6 | 2021.01 | M | 78 | No | II-III | Alive |
| 7 | 2021.03 | F | 56 | No | II | Alive |
| 8 | 2021.03 | F | 75 | No | II | Alive |
| 9 | 2021.03 | F | 49 | No | III | Alive |
| 10 | 2021.03 | F | 56 | No | II | Alive |
| 11 | 2021.03 | F | 54 | No | II | Alive |
| 12 | 2021.04 | F | 55 | No | II | Alive |
| 13 | 2021.04 | M | 65 | No | III | Alive |
| 14 | 2021.04 | M | 61 | No | II-III | Alive |
| 15 | 2021.04 | M | 57 | No | II | Alive |
| 16 | 2021.04 | M | 71 | No | II | Alive |
| 17 | 2021.05 | F | 78 | No | II | Alive |
| 18 | 2021.05 | F | 86 | No | II | Alive |
| 19 | 2021.05 | F | 75 | No | III | Alive |
| 20 | 2021.05 | F | 68 | No | III | Alive |
| 21 | 2021.05 | M | 77 | No | II-III | Alive |
| 22 | 2021.05 | M | 67 | No | II | Alive |
| 23 | 2021.06 | M | 87 | No | II | Alive |
| 24 | 2021.06 | F | 65 | No | II | Alive |
| 25 | 2021.06 | F | 47 | No | III | Alive |
| 26 | 2021.06 | M | 39 | No | II | Alive |
| 27 | 2021.06 | F | 76 | No | II | Alive |
| 28 | 2021.06 | M | 65 | No | III | Alive |
| 29 | 2021.09 | M | 55 | No | II-III | Alive |
| 30 | 2021.09 | M | 43 | No | II | Alive |

Supplementary Table 3 Gene ontology (GO) enrichment analysis results

| **ONTOLOGY** | **ID** | **Description** | **pvalue** |
| --- | --- | --- | --- |
| BP | GO:0060579 | ventral spinal cord interneuron fate commitment | 4.19E-05 |
| BP | GO:0060581 | cell fate commitment involved in pattern specification | 4.19E-05 |
| BP | GO:0021514 | ventral spinal cord interneuron differentiation | 5.93E-05 |
| BP | GO:0021513 | spinal cord dorsal/ventral patterning | 0.000103158 |
| BP | GO:0021511 | spinal cord patterning | 0.000129568 |
| BP | GO:0048665 | neuron fate specification | 0.00026483 |
| BP | GO:0021517 | ventral spinal cord development | 0.000675563 |
| BP | GO:0021515 | cell differentiation in spinal cord | 0.00087701 |
| BP | GO:0048663 | neuron fate commitment | 0.00126925 |
| BP | GO:0019731 | antibacterial humoral response | 0.00135609 |
| BP | GO:0009953 | dorsal/ventral pattern formation | 0.002149973 |
| BP | GO:0001708 | cell fate specification | 0.00279642 |
| BP | GO:0021510 | spinal cord development | 0.003523176 |
| BP | GO:0019730 | antimicrobial humoral response | 0.007107361 |
| BP | GO:0072503 | cellular divalent inorganic cation homeostasis | 0.008537753 |
| BP | GO:1900003 | regulation of serine-type endopeptidase activity | 0.008978497 |
| BP | GO:1902571 | regulation of serine-type peptidase activity | 0.008978497 |
| BP | GO:0072507 | divalent inorganic cation homeostasis | 0.009493425 |
| BP | GO:0051673 | membrane disruption in other organism | 0.009872162 |
| BP | GO:0021953 | central nervous system neuron differentiation | 0.010973333 |
| BP | GO:0010950 | positive regulation of endopeptidase activity | 0.011449289 |
| BP | GO:0010469 | regulation of signaling receptor activity | 0.012180162 |
| BP | GO:0045986 | negative regulation of smooth muscle contraction | 0.012548607 |
| BP | GO:0010273 | detoxification of copper ion | 0.013439241 |
| BP | GO:0050965 | detection of temperature stimulus involved in sensory perception of pain | 0.013439241 |
| BP | GO:1990169 | stress response to copper ion | 0.013439241 |
| BP | GO:0010952 | positive regulation of peptidase activity | 0.013702114 |
| BP | GO:0045779 | negative regulation of bone resorption | 0.01432912 |
| BP | GO:0098962 | regulation of postsynaptic neurotransmitter receptor activity | 0.01432912 |
| BP | GO:0003085 | negative regulation of systemic arterial blood pressure | 0.015218242 |
| BP | GO:0050961 | detection of temperature stimulus involved in sensory perception | 0.015218242 |
| BP | GO:0061687 | detoxification of inorganic compound | 0.015218242 |
| BP | GO:0046851 | negative regulation of bone remodeling | 0.01610661 |
| BP | GO:0097501 | stress response to metal ion | 0.01610661 |
| BP | GO:1901317 | regulation of flagellated sperm motility | 0.01610661 |
| BP | GO:0002089 | lens morphogenesis in camera-type eye | 0.016994223 |
| BP | GO:0030595 | leukocyte chemotaxis | 0.017268016 |
| BP | GO:0098657 | import into cell | 0.017848403 |
| BP | GO:0034104 | negative regulation of tissue remodeling | 0.017881083 |
| BP | GO:0098703 | calcium ion import across plasma membrane | 0.017881083 |
| BP | GO:0016048 | detection of temperature stimulus | 0.01876719 |
| BP | GO:0099633 | protein localization to postsynaptic specialization membrane | 0.01876719 |
| BP | GO:0099645 | neurotransmitter receptor localization to postsynaptic specialization membrane | 0.01876719 |
| BP | GO:1902656 | calcium ion import into cytosol | 0.01876719 |
| BP | GO:0045932 | negative regulation of muscle contraction | 0.019652544 |
| ...... | ...... | ...... | ...... |
| MF | GO:0005245 | voltage-gated calcium channel activity | 0.040035247 |
| MF | GO:0005246 | calcium channel regulator activity | 0.040927359 |

Supplementary Table 4 Kyoto encyclopedia of genes and genomes (KEGG) pathway enrichment analysis results

| **ONTOLOGY** | **ID** | **Description** | **pvalue** |
| --- | --- | --- | --- |
| KEGG | hsa04978 | Mineral absorption | 0.001107509 |

Supplementary Table 5 Gene set enrichment analysis (GSEA) results

| **ID** | **Description** | **ES** | **pvalue** |
| --- | --- | --- | --- |
| GOBP_ACTIVATION_OF_IMMUNE_RESPONSE | GOBP_ACTIVATION_OF_IMMUNE_RESPONSE | 0.481040126 | 1.00E-10 |
| GOBP_ADAPTIVE_IMMUNE_RESPONSE | GOBP_ADAPTIVE_IMMUNE_RESPONSE | 0.587030859 | 1.00E-10 |
| GOBP_CELLULAR_RESPONSE_TO_BIOTIC_STIMULUS | GOBP_CELLULAR_RESPONSE_TO_BIOTIC_STIMULUS | 0.548753591 | 1.00E-10 |
| GOBP_CELLULAR_RESPONSE_TO_MOLECULE_OF_BACTERIAL_ORIGIN | GOBP_CELLULAR_RESPONSE_TO_MOLECULE_OF_BACTERIAL_ORIGIN | 0.571637353 | 1.00E-10 |
| GOBP_DEFENSE_RESPONSE_TO_VIRUS | GOBP_DEFENSE_RESPONSE_TO_VIRUS | 0.561784184 | 1.00E-10 |
| GOBP_IMMUNE_RESPONSE_REGULATING_SIGNALING_PATHWAY | GOBP_IMMUNE_RESPONSE_REGULATING_SIGNALING_PATHWAY | 0.540155261 | 1.00E-10 |
| GOBP_LEUKOCYTE_CELL_CELL_ADHESION | GOBP_LEUKOCYTE_CELL_CELL_ADHESION | 0.519606562 | 1.00E-10 |
| GOBP_LYMPHOCYTE_MEDIATED_IMMUNITY | GOBP_LYMPHOCYTE_MEDIATED_IMMUNITY | 0.543857772 | 1.00E-10 |
| GOBP_MONONUCLEAR_CELL_DIFFERENTIATION | GOBP_MONONUCLEAR_CELL_DIFFERENTIATION | 0.471540255 | 1.00E-10 |
| GOBP_POSITIVE_REGULATION_OF_CELL_ACTIVATION | GOBP_POSITIVE_REGULATION_OF_CELL_ACTIVATION | 0.540004301 | 1.00E-10 |
| GOBP_POSITIVE_REGULATION_OF_CELL_KILLING | GOBP_POSITIVE_REGULATION_OF_CELL_KILLING | 0.769125456 | 1.00E-10 |
| GOBP_POSITIVE_REGULATION_OF_CYTOKINE_PRODUCTION | GOBP_POSITIVE_REGULATION_OF_CYTOKINE_PRODUCTION | 0.536088881 | 1.00E-10 |
| GOBP_POSITIVE_REGULATION_OF_DEFENSE_RESPONSE | GOBP_POSITIVE_REGULATION_OF_DEFENSE_RESPONSE | 0.510039364 | 1.00E-10 |
| GOBP_POSITIVE_REGULATION_OF_IMMUNE_EFFECTOR_PROCESS | GOBP_POSITIVE_REGULATION_OF_IMMUNE_EFFECTOR_PROCESS | 0.615469149 | 1.00E-10 |
| GOBP_POSITIVE_REGULATION_OF_LEUKOCYTE_CELL_CELL_ADHESION | GOBP_POSITIVE_REGULATION_OF_LEUKOCYTE_CELL_CELL_ADHESION | 0.564403241 | 1.00E-10 |
| GOBP_POSITIVE_REGULATION_OF_LEUKOCYTE_MEDIATED_IMMUNITY | GOBP_POSITIVE_REGULATION_OF_LEUKOCYTE_MEDIATED_IMMUNITY | 0.667432752 | 1.00E-10 |
| GOBP_POSITIVE_REGULATION_OF_RESPONSE_TO_EXTERNAL_STIMULUS | GOBP_POSITIVE_REGULATION_OF_RESPONSE_TO_EXTERNAL_STIMULUS | 0.446023446 | 1.00E-10 |
| GOBP_REGULATION_OF_CELL_KILLING | GOBP_REGULATION_OF_CELL_KILLING | 0.727449803 | 1.00E-10 |
| GOBP_REGULATION_OF_IMMUNE_EFFECTOR_PROCESS | GOBP_REGULATION_OF_IMMUNE_EFFECTOR_PROCESS | 0.52518087 | 1.00E-10 |
| GOBP_REGULATION_OF_INNATE_IMMUNE_RESPONSE | GOBP_REGULATION_OF_INNATE_IMMUNE_RESPONSE | 0.505032349 | 1.00E-10 |
| GOBP_REGULATION_OF_LEUKOCYTE_MEDIATED_CYTOTOXICITY | GOBP_REGULATION_OF_LEUKOCYTE_MEDIATED_CYTOTOXICITY | 0.725373256 | 1.00E-10 |
| GOBP_REGULATION_OF_LEUKOCYTE_MEDIATED_IMMUNITY | GOBP_REGULATION_OF_LEUKOCYTE_MEDIATED_IMMUNITY | 0.627244932 | 1.00E-10 |
| GOBP_REGULATION_OF_LYMPHOCYTE_ACTIVATION | GOBP_REGULATION_OF_LYMPHOCYTE_ACTIVATION | 0.515211802 | 1.00E-10 |
| GOBP_REGULATION_OF_LYMPHOCYTE_MEDIATED_IMMUNITY | GOBP_REGULATION_OF_LYMPHOCYTE_MEDIATED_IMMUNITY | 0.628974716 | 1.00E-10 |
| GOBP_REGULATION_OF_RESPONSE_TO_BIOTIC_STIMULUS | GOBP_REGULATION_OF_RESPONSE_TO_BIOTIC_STIMULUS | 0.50038285 | 1.00E-10 |
| GOBP_REGULATION_OF_T_CELL_ACTIVATION | GOBP_REGULATION_OF_T_CELL_ACTIVATION | 0.550177305 | 1.00E-10 |
| GOBP_RESPONSE_TO_INTERFERON_GAMMA | GOBP_RESPONSE_TO_INTERFERON_GAMMA | 0.635122975 | 1.00E-10 |
| GOBP_RESPONSE_TO_VIRUS | GOBP_RESPONSE_TO_VIRUS | 0.528741345 | 1.00E-10 |
| GOBP_T_CELL_ACTIVATION | GOBP_T_CELL_ACTIVATION | 0.512581995 | 1.00E-10 |
| GOMF_CYTOKINE_RECEPTOR_BINDING | GOMF_CYTOKINE_RECEPTOR_BINDING | 0.573447303 | 1.00E-10 |
| GOBP_LEUKOCYTE_PROLIFERATION | GOBP_LEUKOCYTE_PROLIFERATION | 0.501712504 | 1.57E-10 |
| GOBP_GRANULOCYTE_CHEMOTAXIS | GOBP_GRANULOCYTE_CHEMOTAXIS | 0.647050719 | 2.41E-10 |
| GOBP_REGULATION_OF_LEUKOCYTE_DIFFERENTIATION | GOBP_REGULATION_OF_LEUKOCYTE_DIFFERENTIATION | 0.499161209 | 4.79E-10 |
| GOBP_NEGATIVE_REGULATION_OF_IMMUNE_SYSTEM_PROCESS | GOBP_NEGATIVE_REGULATION_OF_IMMUNE_SYSTEM_PROCESS | 0.454912593 | 6.12E-10 |
| GOBP_ANTIGEN_RECEPTOR_MEDIATED_SIGNALING_PATHWAY | GOBP_ANTIGEN_RECEPTOR_MEDIATED_SIGNALING_PATHWAY | 0.520105704 | 1.02E-09 |
| GOBP_T_CELL_ACTIVATION_INVOLVED_IN_IMMUNE_RESPONSE | GOBP_T_CELL_ACTIVATION_INVOLVED_IN_IMMUNE_RESPONSE | 0.648171635 | 1.08E-09 |
| GOBP_REGULATION_OF_CELL_CELL_ADHESION | GOBP_REGULATION_OF_CELL_CELL_ADHESION | 0.439340174 | 1.35E-09 |
| GOBP_LEUKOCYTE_MEDIATED_CYTOTOXICITY | GOBP_LEUKOCYTE_MEDIATED_CYTOTOXICITY | 0.645960608 | 1.41E-09 |
| GOBP_T_CELL_PROLIFERATION | GOBP_T_CELL_PROLIFERATION | 0.552886532 | 1.51E-09 |
| GOBP_POSITIVE_REGULATION_OF_CELL_CELL_ADHESION | GOBP_POSITIVE_REGULATION_OF_CELL_CELL_ADHESION | 0.499802691 | 1.65E-09 |
| GOBP_POSITIVE_REGULATION_OF_RESPONSE_TO_BIOTIC_STIMULUS | GOBP_POSITIVE_REGULATION_OF_RESPONSE_TO_BIOTIC_STIMULUS | 0.512332465 | 1.74E-09 |
| GOBP_NEUTROPHIL_CHEMOTAXIS | GOBP_NEUTROPHIL_CHEMOTAXIS | 0.659590885 | 1.81E-09 |
| GOBP_GRANULOCYTE_MIGRATION | GOBP_GRANULOCYTE_MIGRATION | 0.595305731 | 1.90E-09 |
| GOBP_RESPONSE_TO_MOLECULE_OF_BACTERIAL_ORIGIN | GOBP_RESPONSE_TO_MOLECULE_OF_BACTERIAL_ORIGIN | 0.476131546 | 2.23E-09 |
| GOBP_T_CELL_DIFFERENTIATION | GOBP_T_CELL_DIFFERENTIATION | 0.507608769 | 2.35E-09 |
| GOBP_LYMPHOCYTE_ACTIVATION_INVOLVED_IN_IMMUNE_RESPONSE | GOBP_LYMPHOCYTE_ACTIVATION_INVOLVED_IN_IMMUNE_RESPONSE | 0.551275411 | 2.45E-09 |
| GOBP_CELL_KILLING | GOBP_CELL_KILLING | 0.558031567 | 2.61E-09 |
| GOBP_REGULATION_OF_ADAPTIVE_IMMUNE_RESPONSE | GOBP_REGULATION_OF_ADAPTIVE_IMMUNE_RESPONSE | 0.575885277 | 2.73E-09 |
| GOBP_POSITIVE_REGULATION_OF_CELL_ADHESION | GOBP_POSITIVE_REGULATION_OF_CELL_ADHESION | 0.438472412 | 4.39E-09 |
| GOBP_ADAPTIVE_IMMUNE_RESPONSE_BASED_ON_SOMATIC_RECOMBINATION_OF_IMMUNE_RECEPTORS_BUILT_FROM_IMMUNOGLOBULIN_SUPERFAMILY_DOMAINS | GOBP_ADAPTIVE_IMMUNE_RESPONSE_BASED_ON_SOMATIC_RECOMBINATION_OF_IMMUNE_RECEPTORS_BUILT_FROM_IMMUNOGLOBULIN_SUPERFAMILY_DOMAINS | 0.502223515 | 4.80E-09 |
| GOBP_REGULATION_OF_NATURAL_KILLER_CELL_MEDIATED_IMMUNITY | GOBP_REGULATION_OF_NATURAL_KILLER_CELL_MEDIATED_IMMUNITY | 0.774983187 | 5.05E-09 |
| GOBP_REGULATION_OF_LEUKOCYTE_PROLIFERATION | GOBP_REGULATION_OF_LEUKOCYTE_PROLIFERATION | 0.515621737 | 6.08E-09 |
| GOCC_TERTIARY_GRANULE_MEMBRANE | GOCC_TERTIARY_GRANULE_MEMBRANE | 0.700972623 | 6.61E-09 |
| GOBP_MYELOID_LEUKOCYTE_MIGRATION | GOBP_MYELOID_LEUKOCYTE_MIGRATION | 0.518414977 | 7.06E-09 |
| GOBP_MACROPHAGE_ACTIVATION | GOBP_MACROPHAGE_ACTIVATION | 0.645537183 | 1.01E-08 |
| GOBP_REGULATION_OF_LYMPHOCYTE_DIFFERENTIATION | GOBP_REGULATION_OF_LYMPHOCYTE_DIFFERENTIATION | 0.542082813 | 1.42E-08 |
| GOBP_REGULATION_OF_T_CELL_MEDIATED_IMMUNITY | GOBP_REGULATION_OF_T_CELL_MEDIATED_IMMUNITY | 0.683170682 | 1.58E-08 |
| GOBP_REGULATION_OF_HEMOPOIESIS | GOBP_REGULATION_OF_HEMOPOIESIS | 0.443674334 | 2.00E-08 |
| GOMF_CYTOKINE_ACTIVITY | GOMF_CYTOKINE_ACTIVITY | 0.503491276 | 2.08E-08 |
| GOBP_NATURAL_KILLER_CELL_MEDIATED_IMMUNITY | GOBP_NATURAL_KILLER_CELL_MEDIATED_IMMUNITY | 0.706699416 | 2.27E-08 |
| GOBP_REGULATION_OF_INFLAMMATORY_RESPONSE | GOBP_REGULATION_OF_INFLAMMATORY_RESPONSE | 0.457251793 | 2.72E-08 |
| GOBP_POSITIVE_REGULATION_OF_T_CELL_PROLIFERATION | GOBP_POSITIVE_REGULATION_OF_T_CELL_PROLIFERATION | 0.637992047 | 2.73E-08 |
| GOBP_NEUTROPHIL_MIGRATION | GOBP_NEUTROPHIL_MIGRATION | 0.594461044 | 3.46E-08 |
| GOBP_T_CELL_RECEPTOR_SIGNALING_PATHWAY | GOBP_T_CELL_RECEPTOR_SIGNALING_PATHWAY | 0.519746664 | 4.62E-08 |
| GOBP_RESPONSE_TO_CHEMOKINE | GOBP_RESPONSE_TO_CHEMOKINE | 0.643650361 | 4.89E-08 |
| GOBP_ALPHA_BETA_T_CELL_ACTIVATION | GOBP_ALPHA_BETA_T_CELL_ACTIVATION | 0.551481005 | 5.73E-08 |
| GOBP_LYMPHOCYTE_COSTIMULATION | GOBP_LYMPHOCYTE_COSTIMULATION | 0.702661195 | 6.69E-08 |
| GOBP_CELL_CHEMOTAXIS | GOBP_CELL_CHEMOTAXIS | 0.461064107 | 7.01E-08 |
| GOCC_TERTIARY_GRANULE | GOCC_TERTIARY_GRANULE | 0.540452316 | 7.90E-08 |
| GOMF_CARBOHYDRATE_BINDING | GOMF_CARBOHYDRATE_BINDING | 0.475012466 | 8.22E-08 |
| GOBP_T_CELL_MEDIATED_IMMUNITY | GOBP_T_CELL_MEDIATED_IMMUNITY | 0.620861717 | 8.37E-08 |
| GOBP_POSITIVE_REGULATION_OF_LEUKOCYTE_PROLIFERATION | GOBP_POSITIVE_REGULATION_OF_LEUKOCYTE_PROLIFERATION | 0.557450306 | 8.50E-08 |
| GOCC_SPECIFIC_GRANULE | GOCC_SPECIFIC_GRANULE | 0.543575877 | 8.57E-08 |
| GOBP_NEGATIVE_REGULATION_OF_CYTOKINE_PRODUCTION | GOBP_NEGATIVE_REGULATION_OF_CYTOKINE_PRODUCTION | 0.458848787 | 1.13E-07 |
| GOBP_INTERLEUKIN_10_PRODUCTION | GOBP_INTERLEUKIN_10_PRODUCTION | 0.71693402 | 1.31E-07 |
| GOBP_NEGATIVE_REGULATION_OF_LYMPHOCYTE_ACTIVATION | GOBP_NEGATIVE_REGULATION_OF_LYMPHOCYTE_ACTIVATION | 0.547922545 | 1.47E-07 |
| GOBP_REGULATION_OF_PRODUCTION_OF_MOLECULAR_MEDIATOR_OF_IMMUNE_RESPONSE | GOBP_REGULATION_OF_PRODUCTION_OF_MOLECULAR_MEDIATOR_OF_IMMUNE_RESPONSE | 0.558045472 | 1.79E-07 |
| GOBP_ANTIGEN_PROCESSING_AND_PRESENTATION | GOBP_ANTIGEN_PROCESSING_AND_PRESENTATION | 0.474672287 | 1.96E-07 |
| GOBP_T_CELL_DIFFERENTIATION_INVOLVED_IN_IMMUNE_RESPONSE | GOBP_T_CELL_DIFFERENTIATION_INVOLVED_IN_IMMUNE_RESPONSE | 0.665250864 | 2.10E-07 |
| GOBP_RESPONSE_TO_INTERLEUKIN_1 | GOBP_RESPONSE_TO_INTERLEUKIN_1 | 0.502553968 | 2.14E-07 |
| GOBP_LEUKOCYTE_CHEMOTAXIS | GOBP_LEUKOCYTE_CHEMOTAXIS | 0.487535083 | 2.58E-07 |
| KEGG_CYTOKINE_CYTOKINE_RECEPTOR_INTERACTION | KEGG_CYTOKINE_CYTOKINE_RECEPTOR_INTERACTION | 0.608427149 | 1.00E-10 |
| KEGG_GRAFT_VERSUS_HOST_DISEASE | KEGG_GRAFT_VERSUS_HOST_DISEASE | 0.862923716 | 1.00E-10 |
| KEGG_LEISHMANIA_INFECTION | KEGG_LEISHMANIA_INFECTION | 0.758405915 | 1.00E-10 |
| KEGG_NATURAL_KILLER_CELL_MEDIATED_CYTOTOXICITY | KEGG_NATURAL_KILLER_CELL_MEDIATED_CYTOTOXICITY | 0.657605486 | 1.00E-10 |
| KEGG_AUTOIMMUNE_THYROID_DISEASE | KEGG_AUTOIMMUNE_THYROID_DISEASE | 0.784756822 | 2.81E-10 |
| KEGG_ANTIGEN_PROCESSING_AND_PRESENTATION | KEGG_ANTIGEN_PROCESSING_AND_PRESENTATION | 0.709700093 | 3.44E-10 |
| KEGG_ALLOGRAFT_REJECTION | KEGG_ALLOGRAFT_REJECTION | 0.817545161 | 8.57E-09 |
| KEGG_JAK_STAT_SIGNALING_PATHWAY | KEGG_JAK_STAT_SIGNALING_PATHWAY | 0.557752819 | 4.84E-08 |
| KEGG_TYPE_I_DIABETES_MELLITUS | KEGG_TYPE_I_DIABETES_MELLITUS | 0.767252821 | 1.38E-07 |
| KEGG_HEMATOPOIETIC_CELL_LINEAGE | KEGG_HEMATOPOIETIC_CELL_LINEAGE | 0.640939891 | 2.58E-07 |
| KEGG_TOLL_LIKE_RECEPTOR_SIGNALING_PATHWAY | KEGG_TOLL_LIKE_RECEPTOR_SIGNALING_PATHWAY | 0.590851081 | 1.57E-06 |
| KEGG_INTESTINAL_IMMUNE_NETWORK_FOR_IGA_PRODUCTION | KEGG_INTESTINAL_IMMUNE_NETWORK_FOR_IGA_PRODUCTION | 0.710031497 | 4.27E-06 |
| KEGG_T_CELL_RECEPTOR_SIGNALING_PATHWAY | KEGG_T_CELL_RECEPTOR_SIGNALING_PATHWAY | 0.562096607 | 5.30E-06 |
| KEGG_NOD_LIKE_RECEPTOR_SIGNALING_PATHWAY | KEGG_NOD_LIKE_RECEPTOR_SIGNALING_PATHWAY | 0.644651389 | 6.23E-06 |
| KEGG_CHEMOKINE_SIGNALING_PATHWAY | KEGG_CHEMOKINE_SIGNALING_PATHWAY | 0.47920795 | 9.82E-06 |
| KEGG_VIRAL_MYOCARDITIS | KEGG_VIRAL_MYOCARDITIS | 0.588697799 | 0.000127359 |
| KEGG_CYTOSOLIC_DNA_SENSING_PATHWAY | KEGG_CYTOSOLIC_DNA_SENSING_PATHWAY | 0.610343228 | 0.000314609 |
| KEGG_SYSTEMIC_LUPUS_ERYTHEMATOSUS | KEGG_SYSTEMIC_LUPUS_ERYTHEMATOSUS | 0.468892976 | 0.000544072 |
| KEGG_ASTHMA | KEGG_ASTHMA | 0.701699002 | 0.000803964 |
| KEGG_PROTEASOME | KEGG_PROTEASOME | 0.566365784 | 0.005889591 |
| KEGG_APOPTOSIS | KEGG_APOPTOSIS | 0.439162267 | 0.012496112 |
| KEGG_CELL_ADHESION_MOLECULES_CAMS | KEGG_CELL_ADHESION_MOLECULES_CAMS | 0.39265717 | 0.015860685 |
| KEGG_DRUG_METABOLISM_CYTOCHROME_P450 | KEGG_DRUG_METABOLISM_CYTOCHROME_P450 | -0.516236023 | 0.01894448 |
| KEGG_AMINO_SUGAR_AND_NUCLEOTIDE_SUGAR_METABOLISM | KEGG_AMINO_SUGAR_AND_NUCLEOTIDE_SUGAR_METABOLISM | 0.525229807 | 0.019942025 |
| KEGG_BETA_ALANINE_METABOLISM | KEGG_BETA_ALANINE_METABOLISM | 0.604589484 | 0.020544798 |
| KEGG_CARDIAC_MUSCLE_CONTRACTION | KEGG_CARDIAC_MUSCLE_CONTRACTION | -0.500445004 | 0.023749889 |
| KEGG_CALCIUM_SIGNALING_PATHWAY | KEGG_CALCIUM_SIGNALING_PATHWAY | -0.429804361 | 0.024273753 |
| KEGG_TASTE_TRANSDUCTION | KEGG_TASTE_TRANSDUCTION | -0.55177169 | 0.025171261 |
| KEGG_CELL_CYCLE | KEGG_CELL_CYCLE | 0.405760594 | 0.025283159 |
| KEGG_METABOLISM_OF_XENOBIOTICS_BY_CYTOCHROME_P450 | KEGG_METABOLISM_OF_XENOBIOTICS_BY_CYTOCHROME_P450 | -0.509572164 | 0.032618241 |
| KEGG_TYROSINE_METABOLISM | KEGG_TYROSINE_METABOLISM | -0.558891966 | 0.033488385 |
| KEGG_DILATED_CARDIOMYOPATHY | KEGG_DILATED_CARDIOMYOPATHY | -0.479294048 | 0.034626039 |
| KEGG_FC_GAMMA_R_MEDIATED_PHAGOCYTOSIS | KEGG_FC_GAMMA_R_MEDIATED_PHAGOCYTOSIS | 0.40238421 | 0.039209455 |
| KEGG_NEUROACTIVE_LIGAND_RECEPTOR_INTERACTION | KEGG_NEUROACTIVE_LIGAND_RECEPTOR_INTERACTION | -0.391981772 | 0.041176471 |
